# Regarding: ‘prognostic value of sarcopenia in patients with nasopharyngeal carcinoma: a meta-analysis’

**DOI:** 10.1080/07853890.2025.2547095

**Published:** 2025-08-18

**Authors:** Yuan Wu, Xintian Xu, Xin Jin

**Affiliations:** a Department of Radiation Oncology, Hubei Cancer Hospital, Tongji Medical College, Huazhong University of Science and Technology, Wuhan, Hubei, China; b Department of Clinical Nutrition, Hubei Cancer Hospital, Tongji Medical College, Huazhong University of Science and Technology, Wuhan, Hubei, China

Dear Editorial Office

We read with great interest the recent meta-analysis, ‘Prognostic value of sarcopenia in patients with nasopharyngeal carcinoma: a meta-analysis’ published in Annals of Medicine [1]. This work represents the first meta-analysis to consolidate evidence on sarcopenia as a prognostic marker in nasopharyngeal carcinoma (NPC). The authors concluded that sarcopenia was associated with poor overall survival, progression-free survival and higher T-stage in patients with NPC. These findings offer important evidence supporting integrating body-composition assessment into routine NPC care. Nevertheless, we call attention to some shortcomings that warrant consideration, as they meaningfully influence the interpretation of results and may have been insufficiently addressed.

First, although the aim of the meta-analysis was to evaluate the prognostic impact of sarcopenia on NPC, all included studies diagnosed sarcopenia solely based on muscle area or CT-defined skeletal muscle index (SMI). According to current consensus, the diagnosis of sarcopenia requires both reduced skeletal muscle mass and impaired muscle function (strength or physical performance). Therefore, the factor studied in this meta-analysis should be labeled, more precisely, as ‘CT/MRI-defined low muscle mass’ rather than sarcopenia. In addition, the pooled estimates in the meta-analysis reflected the influence of pre-treatment muscle mass on prognosis. Some studies have reported that dynamic changes in muscle area during chemoradiotherapy were independently associated with poorer outcomes [2]. The impact of treatment-associated muscle loss on prognosis should be analyzed and discussed in this meta-analysis.

Second, among the included studies, some studies used SMI determined at the third cervical vertebra (C3) on CT, whereas others used SMI at the third lumbar (L3) level. Current evidence supports that SMI at the L3 level on CT is a reliable surrogate measurement for whole-body muscle mass. However, one study showed that the correlation between L3 and C3 was weak in sarcopenic patients with head and neck cancer. The results indicated that skeletal muscle mass at C3 may not predict whole body muscle mass [3]. This methodological heterogeneity should be explicitly addressed and discussed as an additional limitation of the meta-analysis. Therefore, a dedicated subgroup analysis comparing these two methods is warranted.

Third, several studies have also explored the impact of skeletal muscle loss on chemoradiotherapy toxicity in NPC [4,5]. One prospective study showed that sarcopenia was associated with toxicity and severe gastrointestinal reactions in patients treated with chemoradiotherapy. These studies should be discussed in the manuscript.

Although eight studies were included, several originated from the same institution and cover overlapping enrollment periods. For example, He et al. (16) and Hua et al. (17) appear to draw from the same center during similar time-frames. The patients enrolled in Huang et al. (15) and Liu et al. (18) were also from same hospital. The double-counting of patients could overestimate the precision of pooled hazard ratios. This issue should be discussed in the article.

In summary, this meta-analysis establishes sarcopenia as a promising prognostic biomarker in NPC, but further work addressing the above limitations will be essential. We encourage the authors to update their analysis once prospective, uniformly defined cohorts become available.

## Data Availability

Not applicable as no new data was generated.
